# (2*E*)-3-[4-(Dimethyl­amino)­phen­yl]-1-(2,5-dimethyl-3-thien­yl)prop-2-en-1-one

**DOI:** 10.1107/S1600536810033751

**Published:** 2010-08-25

**Authors:** Abdullah M. Asiri, Salman A. Khan, M. Nawaz Tahir

**Affiliations:** a The Center of Excellence for Advanced Materials Research, King Abdul Aziz University, Jeddah 21589, PO Box 80203, Saudi Arabia; b Department of Chemistry, Faculty of Science, King Abdul Aziz University, Jeddah 21589, PO Box 80203, Saudi Arabia; c Department of Physics, University of Sargodha, Sargodha, Pakistan

## Abstract

The asymmetric unit of the title compound, C_17_H_19_NOS, contains two independent mol­ecules which differ in the dihedral angles between the five- and six-membered rings [12.52 (10) and 4.63 (11)°]. Weak inter­molecular C—H⋯O hydrogen bonds link the two independent mol­ecules into pseudocentrosymmetric dimers. In one mol­ecule, the O atom of the carbonyl group is disordered over two positions in a 0.699 (4):0.301 (4) ratio.

## Related literature

For background and related crystal structures, see: Asiri *et al.* (2010**a* ▶,*b* ▶,c* ▶). For graph-set notation, see: Bernstein *et al.* (1995 ▶).
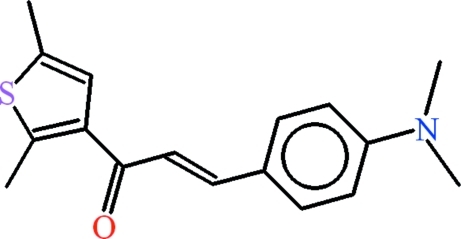


## Experimental

### 

#### Crystal data

C_17_H_19_NOS
*M* *_r_* = 285.40
Triclinic, 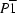

*a* = 7.7665 (2) Å
*b* = 12.8624 (4) Å
*c* = 16.0318 (4) Å
α = 79.917 (1)°
β = 80.029 (2)°
γ = 79.300 (1)°
*V* = 1532.90 (7) Å^3^
*Z* = 4
Mo *K*α radiation
μ = 0.21 mm^−1^
*T* = 296 K
0.32 × 0.23 × 0.20 mm

#### Data collection

Bruker Kappa APEXII CCD diffractometer
Absorption correction: multi-scan (*SADABS*; Bruker, 2005 ▶) *T*_min_ = 0.947, *T*_max_ = 0.962
22632 measured reflections
5536 independent reflections
3543 reflections with *I* > 2σ(*I*)
*R*_int_ = 0.039

#### Refinement

*R*[*F* ^2^ > 2σ(*F* ^2^)] = 0.049
*wR*(*F* ^2^) = 0.156
*S* = 1.02
5536 reflections
373 parameters
H-atom parameters constrained
Δρ_max_ = 0.19 e Å^−3^
Δρ_min_ = −0.23 e Å^−3^

### 

Data collection: *APEX2* (Bruker, 2009 ▶); cell refinement: *SAINT* (Bruker, 2009 ▶); data reduction: *SAINT*; program(s) used to solve structure: *SHELXS97* (Sheldrick, 2008 ▶); program(s) used to refine structure: *SHELXL97* (Sheldrick, 2008 ▶); molecular graphics: *ORTEP-3 for Windows* (Farrugia, 1997 ▶) and *PLATON* (Spek, 2009 ▶); software used to prepare material for publication: *WinGX* (Farrugia, 1999 ▶) and *PLATON*.

## Supplementary Material

Crystal structure: contains datablocks global, I. DOI: 10.1107/S1600536810033751/cv2751sup1.cif

Structure factors: contains datablocks I. DOI: 10.1107/S1600536810033751/cv2751Isup2.hkl

Additional supplementary materials:  crystallographic information; 3D view; checkCIF report

## Figures and Tables

**Table 1:** Hydrogen-bond geometry (Å, °)

*D*—H⋯*A*	*D*—H	H⋯*A*	*D*⋯*A*	*D*—H⋯*A*
C6—H6⋯O2	0.93	2.48	3.275 (3)	143
C19—H19⋯O1*A*	0.93	2.52	3.317 (9)	144
C19—H19⋯O1*B*	0.93	2.48	3.264 (3)	142

**Fig. 1.**
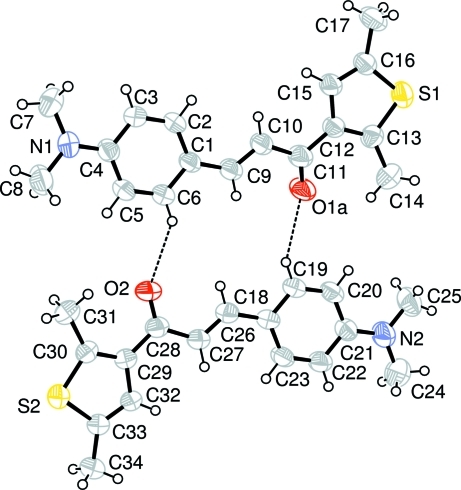
Two independent molecules of (I) with the atomic numbering and 50% probability displacement ellipsoids. Dashed lines denote intermolecular hydrogen bonds. Only major part of the disordered atom (O1A) is shown.

## supplementary crystallographic information

### Comment

In continuation of our structural studies of 2,5-dimethylthiophen-3-yl derivatives (Asiri *et al.*, 2010**a*, b, c*), we present here the crystal structure of the title compound, (I)  (Fig. 1).

The asymmetric unit of (I) contains two independent molecules having different configurations. In one molecule, the phenyl ring A (C1—C6) of 4-(dimethylamino)phenyl, the central group B (C9—C11/O1A) and group C (C12—C17/S1) of 2,5-dimethylthiophen are planar with r. m. s. deviation of 0.0070, 0.0455 and 0.0255 Å, respectively. The dimethylamino group D (C7/N1/C8) is of course planar. The dihedral angle between A/B, A/C, A/D and B/C is 16.29 (39), 12.52 (10), 4.53 (27) and 12.80 (40) (15)°, respectively. In the second molecule, the phenyl ring E (C18—C23) of 4-(dimethylamino)phenyl, the central group F (C26—C28/O2) and group G (C29—C34/S2) of 2,5-dimethylthiophen are planar with r. m. s. deviation of 0.0028, 0.0015 and 0.0317 Å, respectively. The dihedral angle between E/F, E/G and F/G is 8.01 (20), 4.63 (11), and 11.94 (18)°, respectively. The dimethylamino group H (C24/N2/C25) of this molecule is oriented at a dihedral angle of 2.88 (29) ° with its parent phenyl ring. The title compound essentially consists of dimers which are formed due to C—H···O type of intermolecular H-bonding (Table 1, Fig. 1) and complete *R*_2_^2^(14) ring motif (Bernstein *et al.*, 1995).

### Experimental

A solution of 3-acetyl-2,5-dimethythiophene (0.38 g, 2.5 mmol) and N, *N*-dimethylbenzaldehyde (0.37 g, 2.5 mmol) in ethanolic solution of NaOH (3.0 g in 10 ml of methanol) was stirred for 16 h at room temperature. The solution was poured into ice cold water of pH = 2 (pH adjusted by HCl). The solid was separated and dissolved in CH_2_Cl_2_, washed with saturated solution of NaHCO_3_ and evaporated to dryness. The residual was recrystallized from methanol/chloroform to afford light yellow prisms of (I).

Yield: 86%; m.p. 375–376 K.

IR (KBr) \v_max_ cm^-1^: 2979 (C—H_aliphatic_), 1638 (Cδb=O), 1612 (CδbC), 1167 (C—N).

### Refinement

In one independent molecule, the O-atom of carbonyl group is disordered over two set of sites with occupancy ratio of 0.699 (4):0.301 (4). The disordered O-atoms were refined anisotropically with constrained displacement ellipsoids.

The H-atoms were positioned geometrically (C–H = 0.93–0.96 Å) and refined as riding with *U*_iso_(H) = x*U*_eq_(C), where x = 1.5 for methyl and x = 1.2 for aryl H-atoms.

### Crystal data

**Table:**

C_17_H_19_NOS	*Z* = 4
*M**_r_* = 285.40	*F*(000) = 608
Triclinic, *P*1	*D*_x_ = 1.237 Mg m^−^^3^
Hall symbol: -P 1	Mo *K*α radiation, λ = 0.71073 Å
*a* = 7.7665 (2) Å	Cell parameters from 3543 reflections
*b* = 12.8624 (4) Å	θ = 1.6–25.3°
*c* = 16.0318 (4) Å	µ = 0.21 mm^−^^1^
α = 79.917 (1)°	*T* = 296 K
β = 80.029 (2)°	Prism, yellow
γ = 79.300 (1)°	0.32 × 0.23 × 0.20 mm
*V* = 1532.90 (7) Å^3^	

### Data collection

**Table:**

Bruker Kappa APEXII CCD diffractometer	5536 independent reflections
Radiation source: fine-focus sealed tube	3543 reflections with *I* > 2σ(*I*)
graphite	*R*_int_ = 0.039
Detector resolution: 8.10 pixels mm^-1^	θ_max_ = 25.3°, θ_min_ = 1.6°
ω scans	*h* = −9→9
Absorption correction: multi-scan (*SADABS*; Bruker, 2005)	*k* = −15→15
*T*_min_ = 0.947, *T*_max_ = 0.962	*l* = −19→19
22632 measured reflections	

### Refinement

**Table:**

Refinement on *F*^2^	Primary atom site location: structure-invariant direct methods
Least-squares matrix: full	Secondary atom site location: difference Fourier map
*R*[*F*^2^ > 2σ(*F*^2^)] = 0.049	Hydrogen site location: inferred from neighbouring sites
*wR*(*F*^2^) = 0.156	H-atom parameters constrained
*S* = 1.02	*w* = 1/[σ^2^(*F*_o_^2^) + (0.0809*P*)^2^ + 0.1945*P*] where *P* = (*F*_o_^2^ + 2*F*_c_^2^)/3
5536 reflections	(Δ/σ)_max_ < 0.001
373 parameters	Δρ_max_ = 0.19 e Å^−^^3^
0 restraints	Δρ_min_ = −0.23 e Å^−^^3^

### Special details

**Table:**

Geometry. All e.s.d.'s (except the e.s.d. in the dihedral angle between two l.s. planes) are estimated using the full covariance matrix. The cell e.s.d.'s are taken into account individually in the estimation of e.s.d.'s in distances, angles and torsion angles; correlations between e.s.d.'s in cell parameters are only used when they are defined by crystal symmetry. An approximate (isotropic) treatment of cell e.s.d.'s is used for estimating e.s.d.'s involving l.s. planes.
Refinement. Refinement of *F*^2^ against ALL reflections. The weighted *R*-factor *wR* and goodness of fit *S* are based on *F*^2^, conventional *R*-factors *R* are based on *F*, with *F* set to zero for negative *F*^2^. The threshold expression of *F*^2^ > σ(*F*^2^) is used only for calculating *R*-factors(gt) *etc*. and is not relevant to the choice of reflections for refinement. *R*-factors based on *F*^2^ are statistically about twice as large as those based on *F*, and *R*- factors based on ALL data will be even larger.

### Fractional atomic coordinates and isotropic or equivalent isotropic displacement parameters (Å^2^)

**Table:**

	*x*	*y*	*z*	*U*_iso_*/*U*_eq_	Occ. (<1)
S2	0.67761 (9)	0.59564 (5)	0.72204 (4)	0.0656 (2)	
O2	0.4763 (3)	0.71665 (15)	0.46717 (12)	0.0919 (7)	
N2	0.8597 (3)	0.3931 (2)	0.04152 (14)	0.0836 (7)	
C18	0.6929 (3)	0.54644 (19)	0.25944 (15)	0.0533 (6)	
C19	0.6399 (3)	0.5910 (2)	0.18057 (15)	0.0594 (6)	
H19	0.5662	0.6569	0.1760	0.071*	
C20	0.6922 (3)	0.5417 (2)	0.10983 (16)	0.0634 (7)	
H20	0.6527	0.5742	0.0585	0.076*	
C21	0.8042 (3)	0.4429 (2)	0.11278 (15)	0.0609 (6)	
C22	0.8576 (3)	0.3967 (2)	0.19221 (16)	0.0665 (7)	
H22	0.9306	0.3306	0.1971	0.080*	
C23	0.8036 (3)	0.4480 (2)	0.26256 (16)	0.0628 (7)	
H23	0.8422	0.4158	0.3141	0.075*	
C24	0.9775 (4)	0.2907 (3)	0.0452 (2)	0.0990 (10)	
H24A	0.9187	0.2371	0.0827	0.149*	
H24B	1.0828	0.2966	0.0666	0.149*	
H24C	1.0086	0.2709	−0.0111	0.149*	
C25	0.7976 (4)	0.4364 (3)	−0.03922 (18)	0.0934 (10)	
H25A	0.8450	0.5008	−0.0628	0.140*	
H25B	0.6706	0.4520	−0.0304	0.140*	
H25C	0.8360	0.3851	−0.0782	0.140*	
C26	0.6300 (3)	0.60139 (19)	0.33348 (15)	0.0569 (6)	
H26	0.5475	0.6632	0.3248	0.068*	
C27	0.6737 (3)	0.57594 (19)	0.41143 (15)	0.0583 (6)	
H27	0.7601	0.5169	0.4229	0.070*	
C28	0.5897 (3)	0.6381 (2)	0.48031 (16)	0.0600 (6)	
C29	0.6421 (3)	0.60325 (18)	0.56638 (15)	0.0523 (6)	
C30	0.5930 (3)	0.66231 (19)	0.63207 (15)	0.0551 (6)	
C31	0.4827 (3)	0.7713 (2)	0.63494 (18)	0.0718 (7)	
H31A	0.3609	0.7639	0.6541	0.108*	
H31B	0.4939	0.8124	0.5788	0.108*	
H31C	0.5231	0.8071	0.6738	0.108*	
C32	0.7473 (3)	0.50189 (19)	0.59204 (15)	0.0546 (6)	
H32	0.7916	0.4518	0.5551	0.066*	
C33	0.7761 (3)	0.48573 (19)	0.67384 (15)	0.0569 (6)	
C34	0.86743 (13)	0.38708 (5)	0.72324 (4)	0.0717 (7)	
H34A	0.9208	0.3365	0.6851	0.108*	
H34B	0.7826	0.3560	0.7667	0.108*	
H34C	0.9573	0.4057	0.7495	0.108*	
S1	0.33038 (9)	0.95464 (5)	−0.19233 (3)	0.0728 (2)	
O1A	0.37293 (9)	0.78720 (5)	0.08108 (4)	0.0854 (10)	0.699 (4)
O1B	0.44871 (9)	0.80552 (5)	0.06963 (3)	0.0854 (10)	0.301 (4)
N1	0.1145 (2)	1.14648 (8)	0.49058 (4)	0.0711 (6)	
C1	0.23647 (9)	0.97971 (5)	0.27862 (4)	0.0537 (6)	
C2	0.12944 (9)	1.08011 (5)	0.27559 (4)	0.0605 (6)	
H2	0.0858	1.1112	0.2250	0.073*	
C3	0.0866 (3)	1.1342 (2)	0.34461 (15)	0.0620 (6)	
H3	0.0143	1.2006	0.3400	0.074*	
C4	0.1505 (3)	1.0908 (2)	0.42243 (15)	0.0565 (6)	
C5	0.2551 (3)	0.9896 (2)	0.42629 (15)	0.0598 (6)	
H5	0.2980	0.9578	0.4770	0.072*	
C6	0.2954 (3)	0.9364 (2)	0.35693 (15)	0.0604 (6)	
H6	0.3646	0.8689	0.3620	0.072*	
C7	−0.0074 (4)	1.2468 (2)	0.48868 (19)	0.0850 (9)	
H7A	−0.1218	1.2352	0.4808	0.127*	
H7B	0.0359	1.2978	0.4422	0.127*	
H7C	−0.0170	1.2737	0.5418	0.127*	
C8	0.1848 (4)	1.1031 (3)	0.56916 (17)	0.0845 (9)	
H8A	0.3109	1.0830	0.5569	0.127*	
H8B	0.1325	1.0413	0.5960	0.127*	
H8C	0.1578	1.1560	0.6069	0.127*	
C9	0.2893 (3)	0.9220 (2)	0.20630 (15)	0.0630 (7)	
H9	0.3481	0.8525	0.2182	0.076*	
C10	0.2657 (3)	0.9544 (2)	0.12541 (15)	0.0634 (7)	
H10	0.2076	1.0234	0.1101	0.076*	
C11	0.3278 (4)	0.8854 (2)	0.05931 (17)	0.0710 (7)	
C12	0.3116 (3)	0.93130 (19)	−0.03056 (15)	0.0577 (6)	
C13	0.3820 (3)	0.8784 (2)	−0.09877 (16)	0.0618 (6)	
C14	0.4959 (4)	0.7703 (2)	−0.1008 (2)	0.0947 (10)	
H14A	0.4281	0.7207	−0.1125	0.142*	
H14B	0.5361	0.7454	−0.0463	0.142*	
H14C	0.5962	0.7758	−0.1447	0.142*	
C15	0.2189 (4)	1.0352 (2)	−0.05812 (16)	0.0682 (7)	
H15	0.1634	1.0824	−0.0202	0.082*	
C16	0.2183 (3)	1.0591 (2)	−0.14266 (15)	0.0633 (7)	
C17	0.1387 (4)	1.1611 (2)	−0.19338 (18)	0.0881 (9)	
H17A	0.0753	1.2093	−0.1549	0.132*	
H17B	0.0587	1.1454	−0.2272	0.132*	
H17C	0.2312	1.1937	−0.2304	0.132*	

### Atomic displacement parameters (Å^2^)

**Table:**

	*U*^11^	*U*^22^	*U*^33^	*U*^12^	*U*^13^	*U*^23^
S2	0.0735 (4)	0.0660 (5)	0.0564 (4)	−0.0018 (3)	−0.0110 (3)	−0.0153 (3)
O2	0.1119 (15)	0.0755 (13)	0.0746 (13)	0.0380 (12)	−0.0323 (11)	−0.0120 (10)
N2	0.1085 (19)	0.0809 (18)	0.0615 (15)	0.0004 (14)	−0.0194 (13)	−0.0201 (13)
C18	0.0545 (13)	0.0483 (14)	0.0575 (14)	−0.0085 (11)	−0.0171 (11)	0.0000 (11)
C19	0.0623 (14)	0.0565 (16)	0.0566 (15)	−0.0038 (12)	−0.0178 (12)	0.0018 (12)
C20	0.0667 (15)	0.0723 (18)	0.0515 (15)	−0.0091 (13)	−0.0226 (12)	0.0017 (13)
C21	0.0693 (15)	0.0609 (17)	0.0547 (15)	−0.0110 (13)	−0.0162 (12)	−0.0065 (13)
C22	0.0795 (17)	0.0541 (16)	0.0639 (16)	0.0043 (13)	−0.0222 (13)	−0.0079 (13)
C23	0.0762 (16)	0.0575 (16)	0.0538 (15)	−0.0021 (13)	−0.0244 (12)	−0.0003 (12)
C24	0.114 (3)	0.091 (2)	0.092 (2)	0.001 (2)	−0.0127 (19)	−0.0354 (19)
C25	0.115 (2)	0.111 (3)	0.0604 (18)	−0.020 (2)	−0.0224 (17)	−0.0192 (17)
C26	0.0607 (14)	0.0495 (15)	0.0594 (15)	−0.0054 (11)	−0.0147 (11)	−0.0022 (12)
C27	0.0600 (14)	0.0530 (15)	0.0590 (15)	0.0012 (12)	−0.0146 (12)	−0.0053 (12)
C28	0.0616 (14)	0.0522 (15)	0.0640 (16)	−0.0001 (12)	−0.0138 (12)	−0.0073 (13)
C29	0.0525 (12)	0.0469 (14)	0.0553 (14)	−0.0026 (11)	−0.0091 (10)	−0.0066 (11)
C30	0.0529 (13)	0.0506 (15)	0.0595 (15)	−0.0050 (11)	−0.0042 (11)	−0.0099 (12)
C31	0.0766 (17)	0.0560 (16)	0.0794 (18)	0.0037 (13)	−0.0093 (14)	−0.0180 (14)
C32	0.0576 (13)	0.0484 (14)	0.0547 (14)	0.0027 (11)	−0.0101 (11)	−0.0090 (11)
C33	0.0580 (13)	0.0548 (15)	0.0550 (15)	−0.0013 (11)	−0.0107 (11)	−0.0057 (12)
C34	0.0770 (17)	0.0696 (18)	0.0632 (16)	0.0029 (14)	−0.0205 (13)	−0.0012 (14)
S1	0.0879 (5)	0.0797 (5)	0.0505 (4)	−0.0107 (4)	−0.0075 (3)	−0.0141 (3)
O1A	0.139 (3)	0.0468 (13)	0.0649 (14)	0.0062 (15)	−0.0284 (15)	−0.0026 (11)
O1B	0.139 (3)	0.0468 (13)	0.0649 (14)	0.0062 (15)	−0.0284 (15)	−0.0026 (11)
N1	0.0802 (14)	0.0799 (16)	0.0510 (13)	−0.0014 (12)	−0.0086 (10)	−0.0166 (12)
C1	0.0614 (14)	0.0501 (14)	0.0468 (13)	−0.0070 (11)	−0.0121 (11)	0.0026 (11)
C2	0.0676 (15)	0.0631 (17)	0.0454 (14)	0.0004 (12)	−0.0156 (11)	0.0024 (12)
C3	0.0669 (15)	0.0568 (16)	0.0552 (15)	0.0049 (12)	−0.0113 (12)	−0.0026 (12)
C4	0.0552 (13)	0.0636 (16)	0.0480 (14)	−0.0098 (12)	−0.0060 (11)	−0.0022 (12)
C5	0.0658 (15)	0.0645 (17)	0.0453 (14)	−0.0044 (12)	−0.0150 (11)	0.0024 (12)
C6	0.0683 (15)	0.0557 (16)	0.0518 (15)	0.0004 (12)	−0.0131 (12)	−0.0001 (12)
C7	0.0855 (19)	0.085 (2)	0.081 (2)	0.0012 (16)	−0.0016 (15)	−0.0302 (17)
C8	0.095 (2)	0.104 (2)	0.0560 (17)	−0.0147 (18)	−0.0109 (15)	−0.0182 (16)
C9	0.0800 (17)	0.0522 (15)	0.0543 (15)	−0.0032 (13)	−0.0164 (12)	−0.0021 (12)
C10	0.0822 (17)	0.0533 (16)	0.0525 (15)	−0.0024 (13)	−0.0155 (12)	−0.0055 (12)
C11	0.096 (2)	0.0540 (17)	0.0631 (17)	−0.0001 (14)	−0.0238 (14)	−0.0093 (13)
C12	0.0728 (15)	0.0477 (14)	0.0536 (15)	−0.0027 (12)	−0.0170 (12)	−0.0097 (12)
C13	0.0642 (14)	0.0595 (16)	0.0622 (16)	−0.0053 (12)	−0.0105 (12)	−0.0138 (13)
C14	0.104 (2)	0.083 (2)	0.085 (2)	0.0179 (18)	−0.0061 (17)	−0.0237 (17)
C15	0.0938 (19)	0.0575 (17)	0.0518 (15)	0.0045 (14)	−0.0202 (13)	−0.0123 (12)
C16	0.0753 (16)	0.0615 (17)	0.0524 (15)	−0.0063 (13)	−0.0158 (12)	−0.0046 (12)
C17	0.120 (2)	0.076 (2)	0.0632 (18)	−0.0048 (18)	−0.0308 (17)	0.0076 (15)

### Geometric parameters (Å, °)

**Table:**

S2—C30	1.713 (2)	S1—C13	1.709 (3)
S2—C33	1.720 (2)	O1A—C11	1.250 (3)
O2—C28	1.225 (3)	O1B—C11	1.265 (3)
N2—C21	1.373 (3)	N1—C4	1.371 (2)
N2—C25	1.443 (3)	N1—C8	1.441 (3)
N2—C24	1.455 (3)	N1—C7	1.451 (3)
C18—C23	1.391 (3)	C1—C2	1.3980 (9)
C18—C19	1.391 (3)	C1—C6	1.398 (2)
C18—C26	1.453 (3)	C1—C9	1.444 (2)
C19—C20	1.361 (3)	C2—C3	1.369 (3)
C19—H19	0.9300	C2—H2	0.9300
C20—C21	1.399 (3)	C3—C4	1.408 (3)
C20—H20	0.9300	C3—H3	0.9300
C21—C22	1.404 (3)	C4—C5	1.398 (3)
C22—C23	1.370 (3)	C5—C6	1.367 (3)
C22—H22	0.9300	C5—H5	0.9300
C23—H23	0.9300	C6—H6	0.9300
C24—H24A	0.9600	C7—H7A	0.9600
C24—H24B	0.9600	C7—H7B	0.9600
C24—H24C	0.9600	C7—H7C	0.9600
C25—H25A	0.9600	C8—H8A	0.9600
C25—H25B	0.9600	C8—H8B	0.9600
C25—H25C	0.9600	C8—H8C	0.9600
C26—C27	1.321 (3)	C9—C10	1.324 (3)
C26—H26	0.9300	C9—H9	0.9300
C27—C28	1.466 (3)	C10—C11	1.462 (3)
C27—H27	0.9300	C10—H10	0.9300
C28—C29	1.477 (3)	C11—C12	1.476 (3)
C29—C30	1.366 (3)	C12—C13	1.369 (3)
C29—C32	1.439 (3)	C12—C15	1.430 (3)
C30—C31	1.505 (3)	C13—C14	1.505 (4)
C31—H31A	0.9600	C14—H14A	0.9600
C31—H31B	0.9600	C14—H14B	0.9600
C31—H31C	0.9600	C14—H14C	0.9600
C32—C33	1.342 (3)	C15—C16	1.337 (3)
C32—H32	0.9300	C15—H15	0.9300
C33—C34	1.499 (2)	C16—C17	1.505 (3)
C34—H34A	0.9600	C17—H17A	0.9600
C34—H34B	0.9600	C17—H17B	0.9600
C34—H34C	0.9600	C17—H17C	0.9600
S1—C16	1.702 (3)		
			
C30—S2—C33	93.28 (11)	C4—N1—C8	121.07 (17)
C21—N2—C25	122.0 (2)	C4—N1—C7	121.12 (16)
C21—N2—C24	121.2 (2)	C8—N1—C7	117.61 (17)
C25—N2—C24	116.7 (2)	C2—C1—C6	116.15 (11)
C23—C18—C19	116.4 (2)	C2—C1—C9	123.76 (10)
C23—C18—C26	123.4 (2)	C6—C1—C9	120.08 (15)
C19—C18—C26	120.1 (2)	C3—C2—C1	122.24 (10)
C20—C19—C18	122.4 (2)	C3—C2—H2	118.9
C20—C19—H19	118.8	C1—C2—H2	118.9
C18—C19—H19	118.8	C2—C3—C4	121.0 (2)
C19—C20—C21	121.3 (2)	C2—C3—H3	119.5
C19—C20—H20	119.4	C4—C3—H3	119.5
C21—C20—H20	119.4	N1—C4—C5	121.7 (2)
N2—C21—C20	121.7 (2)	N1—C4—C3	121.3 (2)
N2—C21—C22	121.4 (2)	C5—C4—C3	117.0 (2)
C20—C21—C22	116.9 (2)	C6—C5—C4	121.2 (2)
C23—C22—C21	120.9 (2)	C6—C5—H5	119.4
C23—C22—H22	119.6	C4—C5—H5	119.4
C21—C22—H22	119.6	C5—C6—C1	122.4 (2)
C22—C23—C18	122.2 (2)	C5—C6—H6	118.8
C22—C23—H23	118.9	C1—C6—H6	118.8
C18—C23—H23	118.9	N1—C7—H7A	109.5
N2—C24—H24A	109.5	N1—C7—H7B	109.5
N2—C24—H24B	109.5	H7A—C7—H7B	109.5
H24A—C24—H24B	109.5	N1—C7—H7C	109.5
N2—C24—H24C	109.5	H7A—C7—H7C	109.5
H24A—C24—H24C	109.5	H7B—C7—H7C	109.5
H24B—C24—H24C	109.5	N1—C8—H8A	109.5
N2—C25—H25A	109.5	N1—C8—H8B	109.5
N2—C25—H25B	109.5	H8A—C8—H8B	109.5
H25A—C25—H25B	109.5	N1—C8—H8C	109.5
N2—C25—H25C	109.5	H8A—C8—H8C	109.5
H25A—C25—H25C	109.5	H8B—C8—H8C	109.5
H25B—C25—H25C	109.5	C10—C9—C1	129.1 (2)
C27—C26—C18	129.1 (2)	C10—C9—H9	115.5
C27—C26—H26	115.4	C1—C9—H9	115.5
C18—C26—H26	115.4	C9—C10—C11	122.3 (2)
C26—C27—C28	121.9 (2)	C9—C10—H10	118.9
C26—C27—H27	119.1	C11—C10—H10	118.9
C28—C27—H27	119.1	O1A—C11—C10	119.3 (2)
O2—C28—C27	120.7 (2)	O1B—C11—C10	120.9 (2)
O2—C28—C29	120.5 (2)	O1A—C11—C12	121.6 (2)
C27—C28—C29	118.8 (2)	O1B—C11—C12	115.3 (2)
C30—C29—C32	111.6 (2)	C10—C11—C12	118.6 (2)
C30—C29—C28	124.1 (2)	C13—C12—C15	111.0 (2)
C32—C29—C28	124.2 (2)	C13—C12—C11	123.9 (2)
C29—C30—C31	130.2 (2)	C15—C12—C11	125.1 (2)
C29—C30—S2	110.76 (17)	C12—C13—C14	129.6 (2)
C31—C30—S2	119.09 (18)	C12—C13—S1	110.73 (18)
C30—C31—H31A	109.5	C14—C13—S1	119.7 (2)
C30—C31—H31B	109.5	C13—C14—H14A	109.5
H31A—C31—H31B	109.5	C13—C14—H14B	109.5
C30—C31—H31C	109.5	H14A—C14—H14B	109.5
H31A—C31—H31C	109.5	C13—C14—H14C	109.5
H31B—C31—H31C	109.5	H14A—C14—H14C	109.5
C33—C32—C29	114.3 (2)	H14B—C14—H14C	109.5
C33—C32—H32	122.8	C16—C15—C12	114.7 (2)
C29—C32—H32	122.8	C16—C15—H15	122.7
C32—C33—C34	128.5 (2)	C12—C15—H15	122.7
C32—C33—S2	109.98 (18)	C15—C16—C17	128.8 (2)
C34—C33—S2	121.41 (15)	C15—C16—S1	110.20 (19)
C33—C34—H34A	109.5	C17—C16—S1	120.93 (19)
C33—C34—H34B	109.5	C16—C17—H17A	109.5
H34A—C34—H34B	109.5	C16—C17—H17B	109.5
C33—C34—H34C	109.5	H17A—C17—H17B	109.5
H34A—C34—H34C	109.5	C16—C17—H17C	109.5
H34B—C34—H34C	109.5	H17A—C17—H17C	109.5
C16—S1—C13	93.33 (12)	H17B—C17—H17C	109.5
			
C23—C18—C19—C20	0.2 (4)	C1—C2—C3—C4	0.5 (3)
C26—C18—C19—C20	−178.6 (2)	C8—N1—C4—C5	0.5 (4)
C18—C19—C20—C21	−0.5 (4)	C7—N1—C4—C5	−174.2 (2)
C25—N2—C21—C20	−3.8 (4)	C8—N1—C4—C3	−178.1 (2)
C24—N2—C21—C20	179.5 (3)	C7—N1—C4—C3	7.2 (4)
C25—N2—C21—C22	176.3 (3)	C2—C3—C4—N1	177.12 (19)
C24—N2—C21—C22	−0.5 (4)	C2—C3—C4—C5	−1.5 (3)
C19—C20—C21—N2	−179.1 (2)	N1—C4—C5—C6	−177.6 (2)
C19—C20—C21—C22	0.9 (4)	C3—C4—C5—C6	1.1 (4)
N2—C21—C22—C23	179.0 (2)	C4—C5—C6—C1	0.5 (4)
C20—C21—C22—C23	−1.0 (4)	C2—C1—C6—C5	−1.5 (3)
C21—C22—C23—C18	0.7 (4)	C9—C1—C6—C5	177.3 (2)
C19—C18—C23—C22	−0.3 (4)	C2—C1—C9—C10	7.7 (3)
C26—C18—C23—C22	178.4 (2)	C6—C1—C9—C10	−171.1 (3)
C23—C18—C26—C27	6.1 (4)	C1—C9—C10—C11	−180.0 (2)
C19—C18—C26—C27	−175.2 (2)	C9—C10—C11—O1A	14.4 (4)
C18—C26—C27—C28	−176.8 (2)	C9—C10—C11—O1B	−20.8 (4)
C26—C27—C28—O2	−0.5 (4)	C9—C10—C11—C12	−174.1 (2)
C26—C27—C28—C29	178.5 (2)	O1A—C11—C12—C13	−16.1 (4)
O2—C28—C29—C30	−10.8 (4)	O1B—C11—C12—C13	17.9 (4)
C27—C28—C29—C30	170.2 (2)	C10—C11—C12—C13	172.6 (2)
O2—C28—C29—C32	167.6 (2)	O1A—C11—C12—C15	162.9 (2)
C27—C28—C29—C32	−11.4 (4)	O1B—C11—C12—C15	−163.2 (2)
C32—C29—C30—C31	−179.4 (2)	C10—C11—C12—C15	−8.4 (4)
C28—C29—C30—C31	−0.8 (4)	C15—C12—C13—C14	176.9 (3)
C32—C29—C30—S2	1.0 (3)	C11—C12—C13—C14	−4.0 (4)
C28—C29—C30—S2	179.60 (19)	C15—C12—C13—S1	−1.1 (3)
C33—S2—C30—C29	−1.35 (19)	C11—C12—C13—S1	178.0 (2)
C33—S2—C30—C31	179.03 (19)	C16—S1—C13—C12	1.2 (2)
C30—C29—C32—C33	−0.1 (3)	C16—S1—C13—C14	−177.1 (2)
C28—C29—C32—C33	−178.6 (2)	C13—C12—C15—C16	0.5 (3)
C29—C32—C33—C34	175.0 (2)	C11—C12—C15—C16	−178.6 (3)
C29—C32—C33—S2	−0.9 (3)	C12—C15—C16—C17	−178.2 (3)
C30—S2—C33—C32	1.29 (19)	C12—C15—C16—S1	0.4 (3)
C30—S2—C33—C34	−174.97 (18)	C13—S1—C16—C15	−0.9 (2)
C6—C1—C2—C3	1.04 (17)	C13—S1—C16—C17	177.8 (2)
C9—C1—C2—C3	−177.80 (18)		

### Hydrogen-bond geometry (Å, °)

**Table:**

*D*—H···*A*	*D*—H	H···*A*	*D*···*A*	*D*—H···*A*
C6—H6···O2	0.93	2.48	3.275 (3)	143
C19—H19···O1A	0.93	2.52	3.317 (9)	144
C19—H19···O1B	0.93	2.48	3.264 (3)	142
